# The Opioid Crisis: Filling in the Picture

**DOI:** 10.1176/appi.prcp.20180014

**Published:** 2019-04-17

**Authors:** Kimberly A. Yonkers, Mehmet Sofuoglu

**Affiliations:** ^1^ Department of Psychiatry School of Epidemiology and Public Health Department of Obstetrics, Gynecology, and Reproductive Sciences Yale School of Medicine New Haven CT; ^2^ Department of Psychiatry School of Epidemiology and Public Health Yale School of Medicine, and Veterans Affairs Connecticut Healthcare System West Haven CT

**Keywords:** Opioids, Substance Misuse, Opioid Use Disorder

In this first issue of *Psychiatric Research and Clinical Practice*, we focus on the opioid crisis, covering topics that are likely useful for behavioral health and addiction medicine clinicians. These topics include pharmacological and behavioral treatments of opioid use disorder (OUD) and general medical and psychiatric comorbidities (1, 2). In this special issue, we also present less frequently addressed topics, including strategies for treatment engagement of pregnant women with OUD (3) and opioid use in Latin America (4).

The rise in nonmedical use of opioids is likely not due to one factor. The decision and practice of pharmaceutical companies to expand opioid treatment to noncancer patients and the acceptance of this practice by the scientific and medical community, while largely underestimating the addictive properties of opioids, has greatly enlarged the population exposed to opioids. Building on this expanded exposure, importation of illicit opioids has provided a cheaper means for continuing opioid use in the populations that have developed addictions. The widespread availability of highly potent, illicit synthetic opioids, such as fentanyl, has increased the accessibility and lethality of opioids. Much of this background is described in the article by Sofuoglu, De Vito, and Carroll (1). Sofuoglu and colleagues also provide an overview of the main pharmacological treatments for OUD and the clinical challenges of OUD treatment.

The elevated level of psychiatric comorbidity is also addressed by Sofuoglu and colleagues (1). Depression, anxiety disorders, posttraumatic stress disorder, as well as other substance use disorders—including alcohol, cocaine, cannabis, and tobacco use disorders—commonly co‐occur with OUD. Despite their common occurrence, psychiatric comorbidities have received limited attention, especially with regard to their impact on the clinical care of patients with OUD. Sofuoglu and colleagues expand the literature by including strategies that optimize the clinical management of these psychiatric comorbidities (1).

General medical comorbidities are common as well, and psychiatrists can benefit from the review of these conditions by Slawek and colleagues (2). Whereas many medical complications arise from infections related to injection of substances, other complications can occur directly from opioid use, including hypogonadism and sleep‐disordered breathing. Knowledge of these complications is important because these conditions may lead to symptoms that mimic other psychiatric conditions and because they contribute to the overall morbidity of this vulnerable population, which is at risk for numerous general medical and psychiatric conditions. As well, general medical comorbidities are accompanied by pharmacological treatments, and psychotropic medications may interact with other treatments and contribute to complications. For example, the use of serotonin reuptake inhibitors may worsen sleep among those with sleep apnea or other sleep disorders.

In a chilling section from their article on opioid use in Latin America, Pacurucu‐Castillo et al. (4) describe a policy shift by pharmaceutical companies to increase the marketing of opioids in Latin America to compensate for the reduction in opioid prescriptions in the United States. However, the good news reported from these countries is that the rates of opioid use and misuse are much lower in South and Central America than in their North American neighbors. There appears to be a fear among some people in South America around the risk of addiction that has been dubbed “opium phobia.” This fear may be protective against the likelihood of addiction. In addition, the lower rate of opioid addiction may provide lessons on how to avoid or control OUD. The story is still unfolding in Central and South America, and these countries may be able to learn primary prevention strategies from the United States as well.

The Centers for Disease Control and Prevention's “Treating for Two” initiative (5) helped bring focus to the potential problems of medication use in pregnancy. While the initiative addressed many medications, opioid use in pregnancy is an issue of great concern. Indeed, OUD among pregnant women in the United States increased from 1.5 to 6.5 per 1,000 deliveries between 1999 and 2014 (6), leading clinicians, researchers, and patients to question the optimal ways to manage pain and opioid addiction in pregnancy. Dr. Guille and colleagues (3) recognize the critical role patients have in their treatment and present a shared decision‐making tool designed to help obstetrical patients decide what course of treatment (methadone or buprenorphine or medically assisted tapering of these drugs) to pursue for OUD. This tool followed specific guidelines to provide information, generate a risk‐benefit analysis, and offer decisional guidance to help women make decisions about their treatment. The supplement to the Guille et al. article contains the tool that can be used by clinicians who treat pregnant women with OUD.
